# Surgery combined with intra-operative microwaves ablation for the management of colorectal cancer liver metastasis: A case-matched analysis and evaluation of recurrences

**DOI:** 10.3389/fonc.2022.1023301

**Published:** 2022-10-14

**Authors:** Simone Guadagni, Federica Marmorino, Niccolò Furbetta, Martina Carullo, Desirée Gianardi, Matteo Palmeri, Gregorio Di Franco, Annalisa Comandatore, Roberto Moretto, Elisa Cecilia, Giovanni Dima, Gianluca Masi, Chiara Cremolini, Giulio Di Candio, Luca Morelli

**Affiliations:** ^1^ General Surgery Unit, Department of Translational Research and New Technologies in Medicine and Surgery, University of Pisa, Pisa, Italy; ^2^ Unit of Oncology 2, University Hospital of Pisa, Pisa, Italy; ^3^ Department of Translational Research and New Technologies in Medicine, University of Pisa, Pisa, Italy

**Keywords:** liver metastasis, colorectal cancer, microwaves, liver resection, Thermal Ablation

## Abstract

**Background:**

Hepatic resection is the only chance of cure for a subgroup of patients with colorectal cancer liver metastasis. As the oncologic outcomes of intra-operative microwaves ablation combined with hepatic resection still remain uncertain in this setting, we aimed to compare this approach with surgery alone in patient’s candidate to metastases resection with radical intent.

**Methods:**

Using a case-matched methodology based on age, gender, American Society of Anesthesiology score, Body Mass Index, and burden that take in consideration the number and maximum size of lesions, 20 patients undergoing hepatic resection plus intra-operative microwaves (SURG + IMW group) and 20 patients undergoing hepatic resection alone (SURG group), were included. Relapse-free Survival and post-resection Overall Survival were compared between patients of two groups.

**Results:**

At the median follow up of 22.4 ± 17.8, 12/20 patients (60%) in SURG +IMW group and 13/20 patients (65%) in the SURG group experienced liver metastasis recurrence (p=0.774). None of them had recurrence at the same surgical or ablation site of the first hepatic treatment. 7/12 patients in the SURG+IMW group and 7/13 patients in the SURG group underwent at least one further surgical treatment after relapse (p = 1.000). No difference was reported between the two groups in terms of Relapse-free Survival (p = 0.685) and post-resection Overall Survival (p = 0.151). The use of intra-operative microwaves was not an independent factor affecting Relapse-free Survival and post-resection Overall Survival at univariate and multivariate analysis.

**Conclusions:**

Patients with colorectal cancer liver metastasis undergoing surgery plus intra-operative microwaves have similar post-operative results compared with surgery alone group. The choice between the two approaches could be only technical, depending on the site, number, and volume of the metastases. This approach could also be used in patients with liver metastasis relapse who have already undergone hepatic surgery.

## Introduction

Over the last decades, the outcomes of patients with colorectal cancer liver metastases (CRCLM) have greatly improved thanks to innovations in surgical and ablation techniques, more effective systemic therapeutic regimens and the crucial role of a multidisciplinary management, all factors that have allowed to widely extend the indication for surgery with curative intent, even in patients initially defined unresectable (1, 2).

Parenchymal sparing surgery (PSS) has progressively replaced major hepatectomies, becoming the standard of care for patients with CRCLM suitable for surgery, as it has demonstrated advantages in terms of postoperative complications and of liver function preservation, while ensuring similar oncological outcomes (3). Possible drawbacks of this approach may be related to deep-located lesions, which management can be difficult, potentially causing increased blood loss, a sacrifice of a disproportionate amount of parenchyma compared to the size of the lesion, and inevitably prolonging operative time. In this setting, intra-operative thermal-ablation may represent an appealing alternative that can be combined with surgical resection of peripherally located metastases in order to increase the options of treatment for patients with multiple or even bilobar CRCLM.

Nevertheless, the role of surgery combined with intra-operative thermal ablation with curative intent for the treatment of patients affected by CRCLM is still uncertain.

Some studies have reported inferior results of thermal ablation using radiofrequency respect to surgery alone (4–6); however, the possible impact of intra-operative microwaves (IMW) in this specific setting, could be higher than what is currently considered by surgeons.

The present study aims to compare peri-operative and mid-term oncologic outcomes of patients with CRCLM undergoing surgery plus IMW ablation with those of patients undergoing surgery alone, with also a view on the reiterated treatment of hepatic recurrences.

## Materials and methods

### Patients’ selection

We retrospectively analyzed data of all patients with CRCLM undergoing open hepatic resection alone or hepatic resection plus IMW ablation for CRCLM with curative intent at our tertiary care center. Inclusion criteria were the following: i) histologically confirmed diagnosis of CRCLM, ii) patients undergoing hepatic resection or hepatic resection plus IMW with curative intent. Minimally invasive surgery or radio-frequency ablation represented instead exclusion criteria, as well as absence of follow-up and detailed peri-operative information. Patients were then selected by a one-to-one case-matched methodology, where each patient who had undergone surgery plus IMW ablation (SURG+IMW group) was matched with a comparable patient treated with surgical resection alone (SURG group). Matching criteria were the following: age, gender, ASA (American Society of Anesthesiologists) score, BMI (Body Mass Index) and hepatic lesions burden. The hepatic burden was divided into three groups according to the number and maximum size of CRLM: 1-3 lesion and/or maximum size of the biggest lesion of 3 cm (Low burden), 4-10 lesions and/or maximum size of the biggest lesion between 3 and 5 cm (Intermediate burden), more than 10 lesions and/or maximum size of the biggest lesion more than 5 cm (High burden). The study was approved by the Institutional Review Board.

### Surgical procedures

In patients with CRCLM treated with PSS, the decision to use IMW instead of surgically resecting every single lesion mainly depended on its dimension and location. In particular, small (up to 40mm), deep located lesions (especially of the right lobe), or those highly complex to be removed for their location and/or vascular relationship (for instance those located at the hepato-caval confluence), were preferentially treated with IMW ablation. On the contrary, superficial lesions easy to be removed without excessive sacrifice of liver parenchyma were surgically removed. Monolobar deeply located larger lesions (>40mm) and monolobar multiple CRLM were instead indications for major hepatectomies. PSS was the preferred approach every time it was possible. In all patients an intraoperative Ultrasound (US) scan was performed by the operating surgeon. A maximum number of lesions or maximum size *a priori* was not established, but the operation was considered with curative intent based on a case-by-case surgeon’s judgement of feasibility of radical treatment with surgery alone or surgery + IMW, following the described criteria.

For IMW ablation we used microwaves energy device with AMICA™ generator (Hospital Service, Rome, Italy) and 14 G, 150 mm applicators. The tip of the applicator was directed throughout the hepatic parenchyma under real-time US-guide. We generally used a 40-60 Watt with a total of 2 to 5 minutes in a single energy delivery in order to reach a safe coagulative area.

Surgical removal of metastases was performed either with segmentectomy, wedge resection or metastasectomy with the aid of LigaSure™ “Dolphin Tip” (Medtronic, Milan, Italy). Pringle maneuver was not routinely performed, but in relation to lesions size and location. Anatomical major hepatectomies were taken into consideration in selected cases and were performed with the Lortat-Jacob approach.

Pre-surgical chemotherapy was administered according to disease-related characteristics (clinical presentation, tumour burden, resectability tumour sidedness, and tumour biology) and patient-related factors (performance status, age and comorbidity). All patients were considered for surgery in accordance to oncologists at the multidisciplinary discussion based on surgical and oncological criteria. Among patients treated with pre-operative chemotherapy, no one experienced progression disease after pre-surgery therapy as they were not considered optimal candidate for surgical treatment. Reiterated treatment for recurrences was always considered in accordance to oncologists after multidisciplinary discussion, with both the described approaches.

### Data analysis

Pre-operative variables included age, gender, body mass index (BMI), localization of the primary colon cancer, metachronous or synchronous CRCLM, mucinous histological subtype, gene testing in particular *RAS* and *BRAF* mutation, carcinoembryonic antigen (CEA) level at the colorectal diagnosis and before hepatic surgery, chemotherapy regimen, American Society of Anesthesiologists (ASA) score and Eastern cooperative oncology group performance status (ECOG PS). Perioperative data included combined surgery (removal of the primary tumor plus liver surgery) rate, bilobar lesions rate, segments involved, hepatic burden, operative time, and intra-operative complications. Post-operative short-term data included hospital stay, post-operative complications also expressed by Clavien-Dindo classification (7), and 30-day mortality rate. Follow-up information were obtained by clinical examination and radiological imaging and included Relapse-free Survival (RFS) and post-resection Overall Survival (OS). Moreover, any further hepatic recurrence and reiterated surgical treatments were recorded and evaluated. All patients have been followed up by oncologists and discussed by an appropriate multidisciplinary team.

### Statistical analysis

For data analysis, the Chi-square test was used to define associations between categorical factors and surgical groups. Continuous variables with normal distribution were expressed as mean ± standard deviation (SD) and compared using the ANOVA test. Variables with abnormal distribution were expressed as median and compared using the Kruskal- Wallis Test. Survival was compared using Kaplan–Meier curves and log-rank test. Univariate analyses were performed to determine which variables were associated with postoperative mortality and survival; the variables with a p-value <0.1 at the univariate analysis were subjected to multivariate analysis using the Cox regression method and the results were provided in terms of hazard ratio (HR). A p-value ≤ 0.05 was considered statistically significant. The statistical analysis was performed using SPSS (Statistical Production and Service Solution for Windows, SPSS Inc., Chicago, IL, USA), version 24.

## Results

From December 2014 to December 2021, 104 patients underwent hepatic surgery with curative intent for CRCLM at our tertiary center and met the inclusion criteria of the study. From this pool, we extracted the one-to one case-matched study sample consisting in 20 patients for SURG+IMW group and 20 patients for SURG group.

Patients’ characteristics for each group are summarized in **Table 1**, showing similar baseline features, except for a trend towards a higher rate of synchronous treatment of the primary tumor in the SURG+IMW group (90% vs 65%, p=0.058). In particular, in SURG+IMW group hepatic clearance was combined with primary colon resection in eight cases: two right hemicolectomies, three sigmoidectomies and three anterior rectal resections were performed, whereas in the SURG group we contextually performed three right hemicolectomies, one sigmoidectomy and one anterior rectal resection (p=0.311). Fifteen patients in SURG+IMW group and fourteen patients in the SURG group received systemic treatment before surgery (p=0.925). In particular, most of the patients had received chemotherapy (triplet or doublets) in association with biologic agents. Bilobar distribution of metastases was observed in 30% of patients in SURG+IMW group vs 55% in SURG group, p= 0.110), and more than five segments involved were found in 15% of patients in SURG+IMW group vs 10% in SURG group, p= 0.633), without significant differences between the two groups.

**Table 1:** Pre-operative data.

	SURG+IMW-group(n=20)	SURG-group(n=20)	p value
Age (years), mean ± SD	64.7±11.4	65.7±13.8	0.794
Male: Female, n (%)	10:10 (50.0:50.0)	11:9 (55.5:45.5)	0.752
BMI (kg/m2), mean ± SD	24.7±4.5	25.0±3.0	0.808
Right colon: Left colon, n (%)	6:14 (30.0:70.0)	7:13 (35.0:65.0)	0.736
Metachronous: Synchronous, n (%)	2:18 (10.0:90.0)	7:13(35.0:65.0)	0.058
Mucinous cancer, n (%)	5 (25.0)	6 (30.0)	0.723
Gene testing, n (%)			0.620
Wild type (WT)	10 (50.0)	11 (57.9)	
RAS mutation	9 (45.0)	6 (31.6)	
BRAF mutation	1 (5.0)	2 (10.5)	
MSS: MSI, n (%)	19:1 (95.0:5.0)	19:1(95.0:5.0)	1.000
CEA level at diagnosis < 5 ng/mL, n (%)	3:11 (21.4:78.6)	5:6 (45.5:54.5)	0.201
CEA level pre-surgery < 5 ng/mL, n (%)	6:6 (50.0:50.0)	4:6 (40.0:60.0)	0.639
Systemic treatment before surgery, n (%)	15 (75.0)	14 (70.0)	0.925
ASA score, n (%)			0.726
2	4 (20.0)	6 (30.0)	
3	13 (65.0)	12 (60.0)	
4	3 (15.0)	2 (10.0)	
ECOG PS score, n (%)			1.000
0-1	19 (95.0)	19 (95.0)	
2	1 (5.0)	1 (5.0)	

BMI, Body Mass Index; MSS, Micro-Satellite Stable; MSI, Micro-Satellite Instable; ASA score American Society of Anesthesiologists; ECOG PS score, Eastern cooperative oncology group performance status.

Intra-operative data are expressed in **Table 2**. Operative time was 299.4 ± 92.1 min in SURG+IMW group vs 252.4 ± 78.1 min in SURG group (p=0.09). No differences were found between the two groups in of overall complications rate and their severity according to the Clavien-Dindo classification (p=0.225), as well as in mean hospital stay: 9.8 ± 3.3 days for SURG+IMW group vs 13.7 ± 12.4 days for SURG group (p=0.187). No patient required a re-intervention in the post-operative period. In-hospital mortality was registered in one patient of the SURG group who died 27 days after hepatic resection combined with anterior rectal resection, due to sepsis and hepatic failure.

**Table 2:** Intra-operative data.

	SURG+IMW-group (n=20)	SURG-group (n=20)	p value
Combined surgery, n (%)	8 (40.0)	5 (25.0)	0.311
Bilobar lesions, n (%)	6 (30.0)	11 (55.0)	0.110
Segments involved > 5, n (%)	3 (15.0)	2 (10.0)	0.633
Hepatic Burden			1.000
Low (1-3 lesions, ≤ 3 cm diameter)	3 (15.0)	3 (15.0)	
Intermediate (4-10 lesions, ≤ 5 cm diameter)	12 (60.0)	12 (60.0)	
High (>10 lesions, > 5 cm diameter)	5 (25.0)	5 (25.0)	
Operative time (min), mean ± SD	299.4±92.1	252.4±78.1	0.090
Intra-operative complications, n (%)	0 (0.0)	0 (0.0)	1.00

**Figure 1:**
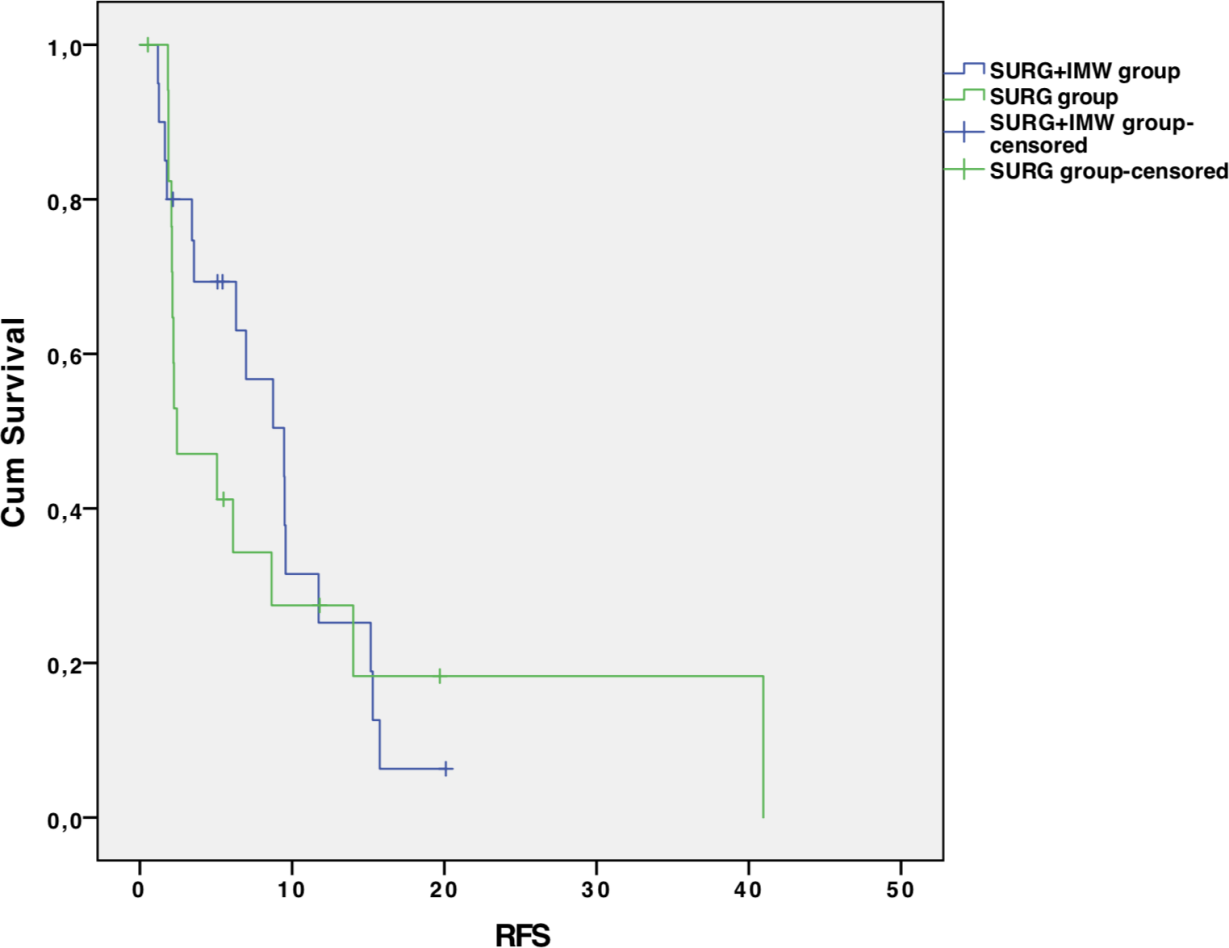
Disease-free survival in the two groups.

**Figure 2:**
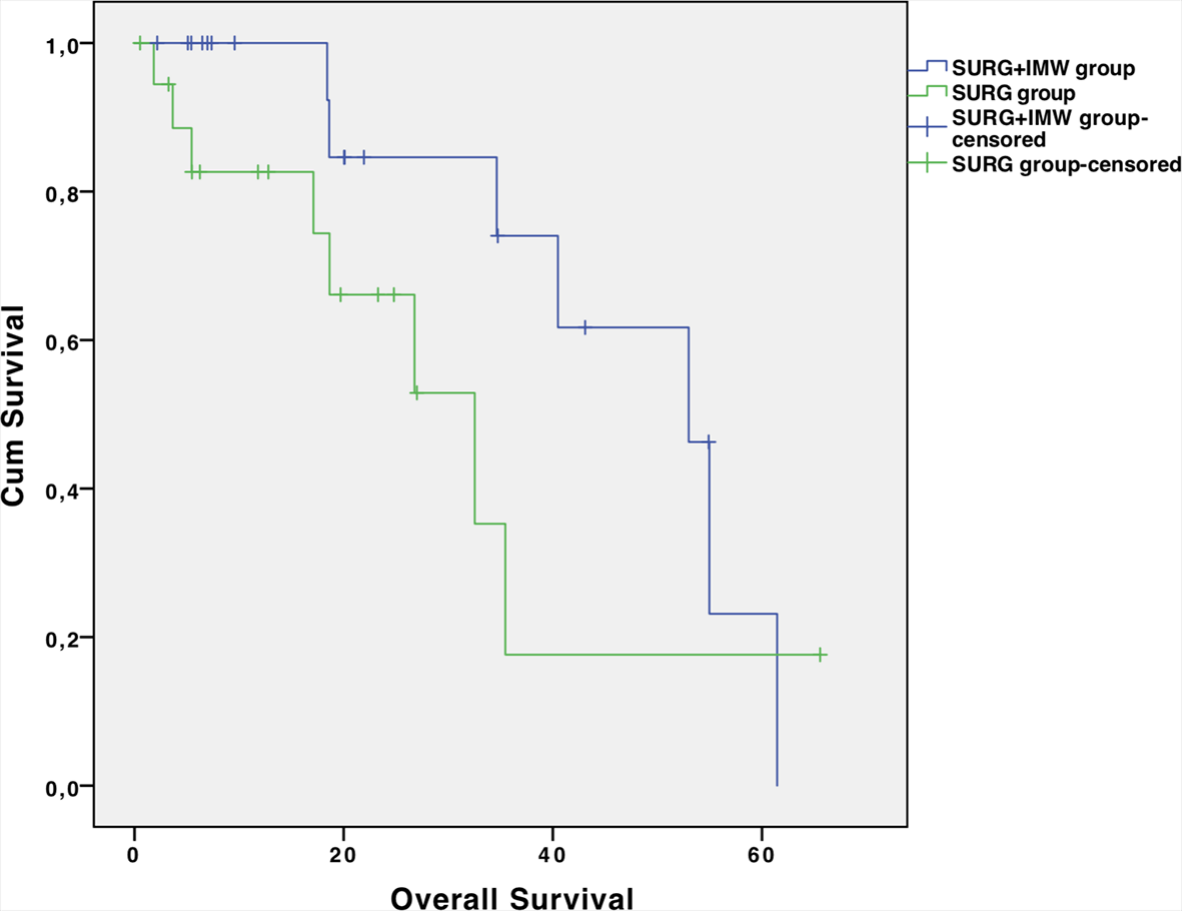
Overall survival in the two groups.

The mean follow-up was 26.0 ± 19.6 months for SURG+IMW group and 18.9 ± 16.0 months for SURG group (p=0.220) (**Table 3**). No significant difference was found in terms of RFS: median RFS was 9.5 months (4.8 – 14.2) for the SURG+IMW group and 2.4 months (0 – 6.3) for the SURG group (HR 1.2; 95% CI 0.56-2.4; p=0.685). No difference was reported between the two groups in terms of post-resection OS: median OS was 53.0 months (39.9 – 66.1) for the SURG+IMW group and 32.5 months (16.7 – 48.2) for the SURG group (HR 2.13; 95% CI 0.74-6.09; p=0.151) (**Figure 1**, **Figure 2**).

**Table 3:** Post-operative data.

	SURG+IMW-group (n=20)	SURG-group (n=20)	p value
Post-operative complications, n (%)	9 (45.0)	14 (70.0)	0.110
Clavien- Dindo grading, n (%)			0.225
0	11 (55.0)	6 (30.0)	
1	1 (5.0)	0 (0.0)	
2	7 (35.0)	11 (55.0)	
3	1 (5.0)	3 (15.0)	
Hospital stays (days), mean ± SD	9.8±3.3	13.7±12.4	0.187
30-days mortality, n (%)	0 (0.0)	1 (5.0)	0.311
Follow up (months), mean ± SD	26.0±19.6	18.9±16.0	0.220
PD post-surgery, n (%)	16 (80.0)	14 (73.7)	0.640
Hepatic recurrence, n (%)	12 (60.0)	13 (65.0)	0.744
Repeat liver resection for recurrence, n (%)	7 (35.0)	7 (35.0)	1.000
RFS (months), median (range)	9.5 (4.8 – 14.2)	2.4 (0 – 6.3)	0.685
OS (months), median (range)	53.0 (39.9 – 66.1)	32.5 (16.7 – 48.2)	0.151

PD, Progression Disease; RFS, Relapse Free survival; OS, Overall Survival.

Twelve patients (60%) in SURG+IMW group and thirteen patients (65%) in SURG group experienced hepatic recurrence after curative treatment (p=0.774). Among them, 7/12 (58.3%) patients of SURG+IMW group and 7/13 (53.7%) patients of SURG group underwent at least one further surgical treatment (p = 1.000). None of them had recurrence at the same surgical or ablation site of the first hepatic treatment.

In univariate analysis, ECOG PS (HR 2.03; 95% CI 0.99-4.18; p=0.054) was significantly associated with shorter RFS, whereas mucinous histology (HR 2.972, 95% CI 0.914-9.667, p=0.07) and ECOG PS (HR 3.344, 95% CI 1.072-10.430, p=0.038) were associated with a reduced post-operative OS.

In the multivariate model, the ECOG PS (HR 4.959; 95% CI 1.385-17.775; p=0.014) and mucinous histology (HR4.113; 95% CI 1.161-14.573; p=0.028) remained significant predictor of post-operative OS (**Table 4**).

**Table 4a:** Univariate and multivariate analysis for OS.

	Univariate Analysis OS			Multivariate Analysis OS		
	p	HR	95% CI	p	HR	95% CI
Surg+IMW vs surgery	0.159	2.128	0.744-6.090			
Right colon vs Left colon	0.840	1.114	0.391-3.169			
Metachronous vs Synchronous	0.511	0.582	0.115-2.930			
Mucinous cancer	**0.070**	2.972	0.914-9.667	**0.028**	4.113	1.161-14.573
WT vs RAS	0.553	1.424	0.443-4.581			
WT vs BRAF	0.783	0.744	0.091-6.106			
MSS vs MSI	0.535	0.043	0.000-895.567			
CEA level at diagnosis < 5 ng/mL	0.851	1.124	0.333-3.789			
CEA level pre-surgery < 5 ng/mL	0.480	0.595	0.141-2.590			
Systemic treatment before surgery	0.146	4.575	0.590-35.459			
ECOG PS score	**0.038**	3.344	1.072-10.430	**0.014**	4.959	1.385-17.775
Bilobar lesions	0.763	1.171	0.420-3.263			

Bold values are statistically significant at univariate and multivariate analysis.

**Table 4b:** Univariate and multivariate analysis for RFS.

		Univariate Analysis DFS
	p	HR	95% CI
Surg+IMW vs surgery	0.685	1.165	0.557-2.437
Right colon vs Left colon	0.505	0.769	0.356-1.663
Metachronous vs Synchronous	0.382	0.688	0.297-1.592
Mucinous cancer	0.428	1.318	0.621-3.072
WT vs RAS	0.282	1.521	0.709-3.264
WT vs BRAF	0.361	2.062	0.436-9.757
MSS vs MSI	0.347	0.042	0.000-31.475
CEA level at diagnosis < 5 ng/mL	0.855	1.090	0.431-2.758
CEA level pre-surgery < 5 ng/mL	0.925	0.953	0.350-2.596
Systemic treatment before surgery	0.992	1.004	0.426-2.366
ECOG PS score	**0.054**	2.032	0.987-4.182
Bilobar lesions	0.307	0.674	0.316-1.436

Focusing on patients who underwent further surgical treatment for CRCLM relapse, in SURG+IMW group 3/7 patients underwent wedge resection, 1/7 underwent wedge resection plus IMW, 1/7 underwent right hepatectomy and 2/7 underwent lateral sectionectomy. One of the patients treated with wedge resection needs a further surgical hepatic clearance for recurrence 10 months later. Among these 7 patients, 2 (28.5%) are still alive with a mean follow up of 31.7 months. In SURG group 7/7 patients underwent wedge resection; three of them (42.8%) are still alive with a mean follow up of 50.0 months.

## Discussion

The surgical treatment of CRCLM in combination with systemic therapies is continuously evolving, leading to a great improvement of oncological outcomes of patients, and even to cure a subgroup of them. Several approaches have been described with the intent of tumor eradication without compromising liver function. Firstly, major hepatectomies and their variants such as portal vein embolization or associating liver partition and portal vein ligation for staged hepatectomy (ALPPS) were widely performed, posing their rationale in an aggressive curative anatomical resection with safe resection margins (8). However, these procedures are characterized by high morbidity and possible tumor progression during the interval period, so that nowadays their indication is much more restricted.

In this scenario, PSS has progressively gained popularity, based on the principle that CRCLM are a systemic disease for which surgery represents an important step of the treatment, but the major address must be organ preservation for further therapies. In fact, this approach can be combined with early systemic treatments and can be also adopted to treat hepatic recurrences which are estimated to affect half of patients within two years after surgery. Several studies have reported on PSS demonstrating better results in terms of postoperative complications and liver function preservation respect to major hepatectomies, while ensuring similar oncological outcomes, thus becoming the surgical treatment of choice for patients with CRCLM (3, 9, 10). However, if PSS is safe and quite simple for superficial lesions, it can become more challenging in case of deeper metastases. Moreover, a high hepatic burden of disease poses some drawbacks related to a possible increase of blood loss, a potential sacrifice of a disproportionate amount of parenchyma respect to the size of the lesion, and surely to a prolonged operative time, all factors that may affect the surgical outcomes. In this scenario with the reported lower morbidity coming from literature (11) intra-operative thermal ablation could play a positive role, representing an appealing alternative option to treat deep-located metastases.

Several studies have described the safety and the potential utility of radiofrequency for CRCLM treatment, but when compared to the surgical approach, it has shown inferior results in terms of survival, either alone or in combination with surgery therefore leading to consider this choice as a fallback, and mostly with palliative intent (4, 6, 11, 12). These findings may be related to the intrinsic limits of the radiofrequency, such as the long time required for each thermo-ablation and the limited size of the of the treated area, that can be surpassed with microwaves.

Confirming this, a recent systematic review (13), concluded that MW ablation for lesions smaller than 3 cm represents a safe and valid option of treatment with curative intent for selected patients with CRCLM, therefore overcoming the widespread concept among surgeons of a less oncological radicality with this alternative approach, at least in selected patients.

However, although this specific ablation technique has been available since twenty years, only few papers have dealt with it in combination with surgery so far (14, 15), and most of them are affected by several bias related to the type of MW device used (mostly currently surpassed), to the heterogeneity of the sample, to the absence of a control group, or to the lack of an oncologic follow-up. This consideration prompted us to review our experience in this field, with a particular attention to the oncological outcomes.

To the best of our knowledge, the present work is the first one that compares surgery plus IMW versus surgery alone with an updated ablation system for the treatment of patients, with the same burden of CRCLM, up to 40 mm for each lesion, using a case match methodology, and with a mid-term oncologic follow-up evaluation.

In our series, similarly, to the peri-operative data, the mid-term survival results were not significantly different between the two groups, and most importantly, the type of intervention did not influence these parameters neither in the univariate nor in the multivariate analysis. Moreover, following our imaging revision, in case of hepatic recurrence, the second relapse did not interest the first surgical or IMW site, reinforcing the concept of efficacy of both treatments.

Hence, our results support that the decision to perform an IMW ablation does not increase the peri-operative morbidity, and does not negatively influence the post-operative survival and the risk of relapse, and therefore should be considered only a technical surgeon’s choice. Indeed, since comparing the same burden of disease we did not register differences in OS and RFS between the two groups, the surgeon should be aware that choosing to treat a small (up to 40 mm), deep metastasis difficult to be removed with MW ablation could be preferable to a more aggressive surgery, as the survival will be not affected by this choice. Instead, in these cases, particularly when facing with multiple metastases, a radical surgery alone is likely to be affected by higher operative times, blood loss, morbidity and mortality, or oblige to an unnecessary liver parenchyma sacrifice.

In this regard, because of its retrospective nature, our series has the limitation that, although the two groups of patients had similar burden of disease and operative risk, the location of the lesions and the surgical complexity of their resection were not exactly comparable and therefore, unlike the oncologic outcomes, the results of surgical outcomes were less meaningful. Nevertheless, although not statistically significant, we registered a trend towards a lower rate of complications and reduced hospitalization in the SURG+IMW group, in line with the propensity score analysis conducted by Xourafas et al. (15) that showed reduced morbidity and length of hospital stay in patients treated with surgery and intra-operative thermal ablation. These results could also be explained by the intuitive observation that in SURG+IMW approach the treatment of deep CRCLM was faster and characterized by a lower parenchymal deep dissection. Another point in favor of IMW ablation is its particularly quick application as, unlike RF which ablation time ranges from 20 to 30 minute for every single lesion, the ablation time of IMW ranges from 2 to 5 minute, therefore allowing multiple treatments without being excessively time consuming. This aspect in our experience has revealed to be particularly important in the treatment of multiple CRCLM, allowing to resect up to 25 superficial metastases and to thermo-ablate up to 26 deep ones in a single patient. Instead, the trend towards a longer operative time registered in SURG+IMW group is probably related to the significantly higher rate of combined interventions (hepatic plus primary cancer resection) in this group.

Thanks to innovations in surgical and ablation techniques and more effective systemic therapeutic regimens and the fundamental role of a multidisciplinary management, the survival of patients with CRCLM is becoming longer and longer, even in case of recurrence, so that oncologists and surgeons are now dealing with a “chronic disease” (16). Surgical resection for second hepatic relapse has been reported to be associated with surgical risk and long-term outcomes similar to those of the first hepatic resection, with a 5-year OS rate ranging from 27 to 45% (17). Only few papers have reported similar results with thermal-ablation in CRCLM recurrences (18). In our study, although surgery was the most used approach for hepatic recurrences, patients who underwent further IMW ablation showed good results, underlining its role as a radical option also in this setting.

Main limitations of the study are the monocentric and retrospective nature, as well as the possible oncologic selection bias related to exclusion of prognostic criteria (i.e., ECOG-PS, *RAS* and *BRAF* mutational status, time to presentation of liver metastases) from matching approach due to small sample size. However, no significant differences were observed between SURG+IMW and SURG groups in terms of prognostic parameters. The limited number of patients included in the study is a relevant shortcoming, but we choose to give more importance to comparability respect to statistical power and therefore we tried to mitigate these limitations by matching patients for hepatic burden of disease and for surgical risk, with the main aim to give indication on the oncological results. Moreover, another limitation could be related to the estimation of hepatic tumor burden as this is another matter of debate with several scores adopted for the evaluation of liver disease load (19, 20). Finally, we included patients enrolled in a long period in which the patient’s selection had undergone important improvements in order to refine the choice of systemic treatment with considerable impact in terms of clinical outcome. While the two cohorts shared homogeneous baseline characteristics, overall population of our work included a diversified spectrum of colon liver metastases patients (resecatable, potentially resectable or initially unresectable) who have received different pre-surgery therapies thus making findings hardly comparable with available data from literature in terms of RFS and OS. After resection of colorectal liver metastases with curative intent, a recent comparative analysis reported a minimal correlation between RFS and OS (21) showing a wide range of time intervals from recurrence to death, thus limiting the value of RFS as a surrogate endpoint for OS. This assumption together with the recent evolution of locoregional and surgical techniques for second hepatic relapse and the availability of active systemic treatments can explain our results in terms of OS.

In conclusion, data emerged from the present case matched series support the use of IMW in association with surgery for the treatment of CRCLM, also in case of hepatic relapse. This approach seems to be not inferior to resection alone in selected patients, and may be particularly indicated in those who have small multiple and deep-located metastases in which we can predict a difficult and time-consuming surgery. IMW ablation should not be considered a worse alternative to surgical resection in patient with multiple CRCLM, but an integrated treatment in a parenchymal sparing approach in which we should balance oncologic outcomes and patient’s safety. This approach could also be used in patients with CRCLM relapse who have already undergone hepatic surgery. Further studies are needed to be more conclusive on the role of IMW ablation in this setting (22).

## Data availability statement

The raw data supporting the conclusions of this article will be made available by the authors, without undue reservation.

## Ethics statement

All procedures performed in studies involving human participants were in accordance with the ethical standards of the institutional and/or national research committee and with the 1964 Helsinki Declaration and its later amendments or comparable ethical standards. Informed consent was obtained from all individual participants involved in the study.

## Author contributions

Study concepts: SG, NF, MP, LM, CC. Study design: MC, GD, AC, NF, GF, SG, FM. Data acquisition: MC, GD, AC, DG, RM, SG, FM. Quality control of data and algorithms: SG, FM, GF, EC, GC, LM. Data analysis and interpretation: GF, SG, FM, MP, EC, GM. Statistical analysis: GF, SG, FM, GD, EC, LM. Manuscript preparation: SG, FM, LM, CC, RM, DG. Manuscript editing: SG, FM, LM, CC, GC, RM, GM. Manuscript review: SG, FM, LM, CC, GC, RM, GM. All authors contributed to the article and approved the submitted version.

## Acknowledgments

The authors thank Arpa Foundation for the support and Sharon Bernadette King for language editing.

## Conflict of interest

The authors declare that the research was conducted in the absence of any commercial or financial relationships that could be construed as a potential conflict of interest.

## Publisher’s note

All claims expressed in this article are solely those of the authors and do not necessarily represent those of their affiliated organizations, or those of the publisher, the editors and the reviewers. Any product that may be evaluated in this article, or claim that may be made by its manufacturer, is not guaranteed or endorsed by the publisher.
